# Initial evaluation rate invariance for Alzheimer’s Disease drug trials after first appointment modification

**DOI:** 10.1002/alz.092240

**Published:** 2025-01-09

**Authors:** Ralph Lee, Yu‐Jay Huoh, Brenda Martinez, Elly Lee

**Affiliations:** ^1^ Irvine Clinical Research, Irvine, CA USA

## Abstract

**Background:**

Recruiting large numbers of study participants for Alzheimer’s Disease (AD) drug trials remains a significant challenge in need of more effective approaches. Advertising can be an effective way to reach large numbers of prospective participants, but can suffer from low attendance rates. This study examined the relationship between the initial behaviors of prospective AD trial participants who did not attend their first scheduled appointment and their overall likelihood of eventually attending an in‐person consultation.

**Method:**

In 2023, 4,043 potential participants were identified through online advertising and scheduled for 5,561 appointments for an in‐person pre‐screen at a commercial research site. Only 45.0% attended their first scheduled appointment. In lieu of attending their first scheduled appointment, 32.3% (n = 1,307) called in to the clinic to modify (i.e. cancel or reschedule) their first scheduled appointment and the remaining 22.7% (n = 915) simply did not attend their first scheduled appointment. Recruiters attempted to reschedule the latter group for another appointment.

**Result:**

Of the 4,043 potential participants who were ever scheduled for an in‐person pre‐screen, 55.8% of these participants eventually attended an appointment, whether during the first or subsequently scheduled appointments.

Of the 1,307 potential participants who called in to modify their first scheduled appointment, 506 of these potential participants were scheduled for a subsequent appointment, with 57.3% of these persons eventually attending. This deviation from the 55.8% overall attendance rate is not statistically significant (p = 0.50).

However, of the 915 potential participants who simply did not attend their first scheduled appointment, 352 were scheduled for a subsequent appointment. Of these 352, only 43.5% eventually attended an appointment. This deviation from both the overall attendance rate and the attendance rate for those who modified their first appointment were highly significant (p < 1e‐4 for both).

**Conclusion:**

Potential AD trial participants who modify their first appointment are adequately engaged in the process, and so not attending their first appointment does not adversely affect their chances of attending a pre‐screen. In contrast, potential participants who simply do not show up are much less likely to ever attend any appointment (almost 25% relative, >13% absolute).

